# Drug-eluting balloon: is it useful?

**DOI:** 10.1186/s43044-020-00116-7

**Published:** 2020-11-11

**Authors:** Sidhi Laksono, Budhi Setianto, Steven Philip Surya

**Affiliations:** 1 Cardiac Catheterization Laboratory, Department of Cardiology and Vascular Medicine, RSUD Pasar Rebo, Jakarta, Indonesia; 2 Faculty of Medicine, Universitas Muhammadiyah Prof. DR. Hamka, Tangerang, Indonesia; 3 Department of Cardiology and Vascular Medicine of National Cardiovascular Center of Harapan Kita, Faculty of Medicine of Universitas Indonesia, Jakarta, Indonesia; 4 Army Hospital Kesdam Jaya Cijantung, Jakarta, Indonesia

**Keywords:** Coronary artery disease, Coronary intervention, Stent, Drug-coated balloon

## Abstract

**Background:**

Coronary artery disease is one of the major issues in the medical world around the globe. The prevalence tends to increase. The use of coronary intervention is one of the ways often used in the management of coronary artery disease due to its satisfying result from earlier studies. Nowadays, there are several different techniques in coronary intervention: balloon vs stent.

**Main body:**

The stent-based vascular interventions are increasingly being used over balloon-based coronary intervention. However, revascularization intervention using stent often have undesirable long-term effects compared to balloon. Besides, stent-based interventions are also considered more expensive, use more complicated techniques, and use more drug regimens. On the other hand, percutaneous coronary intervention techniques using balloons coated by anti-proliferation drugs have begun to be glimpsed by many interventionists. Studies have found many benefits that cannot be given by stent-based intervention therapy.

**Conclusions:**

Angioplasty using percutaneous coronary intervention techniques reveals satisfying result compared to conservative medical treatment. The indication and technique of percutaneous coronary intervention is still evolving until now. Currently, percutaneous coronary intervention using stent, either bare-metal stent or drug-eluting stent, is preferred by interventionist. Nevertheless, recent clinical trial favors the using of drug-eluting balloon for percutaneous coronary intervention in terms of both clinical outcome and complication in several scenarios.

## Background

Since the time when the first-time angioplasty for vascular intervention was introduced, the need of angioplasty for management of artery diseases was increasing. In the increase of angioplasty procedure parallel with its early promising study for the cardiac disease patients, only 5 undesirable events of cardiac death from 169 patients underwent cardiac catheterization after 10 years [1]. The first large trial conducted by the National Heart, Lung, and Blood Institute (NHLBI) which involved 2500 patients with percutaneous transluminal coronary angioplasty (PCTA) showed an unquestionable result from multiple indicators like increase of immediate success rate, decrease in non-fatal myocardial infraction, and emergency coronary artery bypass graft surgery [1]. Stent-based techniques are more common in practice recently, but there are still some limitations to these techniques [2, 3]. Likewise, long-term benefit of the stent-based PCI over drug-eluting balloon techniques is still questionable [2].

## Main body

### Indication

Several previous trial studies showed that angioplasty has greater advantages for relieving symptoms in stable coronary artery disease patients with the no-or-mild symptoms group compared to other medical therapies [4, 5]. However, a randomized trial study discovered no significant difference in cardiac death event, myocardial infraction, and stroke from PCI therapy compared to other optimal medical therapies (OMT) [6]. The decision to undergo PCI for stable angina remains unclear, and it depends on patient-doctor expectations.

Conversely, angioplasty for patient with non-ST elevation myocardial infarct (NSTEMI) should be stratified to the high-risk vs low-risk groups first [4]. The high-risk group was defined by dynamic ST segment changes, elevated troponin-I/troponin-T/CK-MB levels, GRACE score, and other secondary criteria. An obvious benefit of PCI for NSTEMI patients (cardiac death event, myocardial infraction, and refractory angina) was found in patients with the high-risk group [4, 7]. Previous studies showed that immediate intervention has no significant advantages and tends to give more harm in clinically unstable patients [8–10].

More specific indications for PCI were patients with acute coronary syndrome (ACS) and evidence of persistent ST segment elevation (STEMI) or presumed new bundle-branch block. However, primary PCI in STEMI required an interventional cardiologist, short respond time, and well-trained support staff. Even worse, a study from Egypt showed that all patients who were diagnosed with STEMI came into the emergency department at the end of the golden period time or after the golden period time [4]. Another study has demonstrated that pharmacology therapy preceding PCI could prolong the golden period time [11]. Notwithstanding many argumentations about the cutoff of the extended period, the longest time PCI delay was 180 min [12].

### Pathophysiology of restenosis

**Fig. 1:**
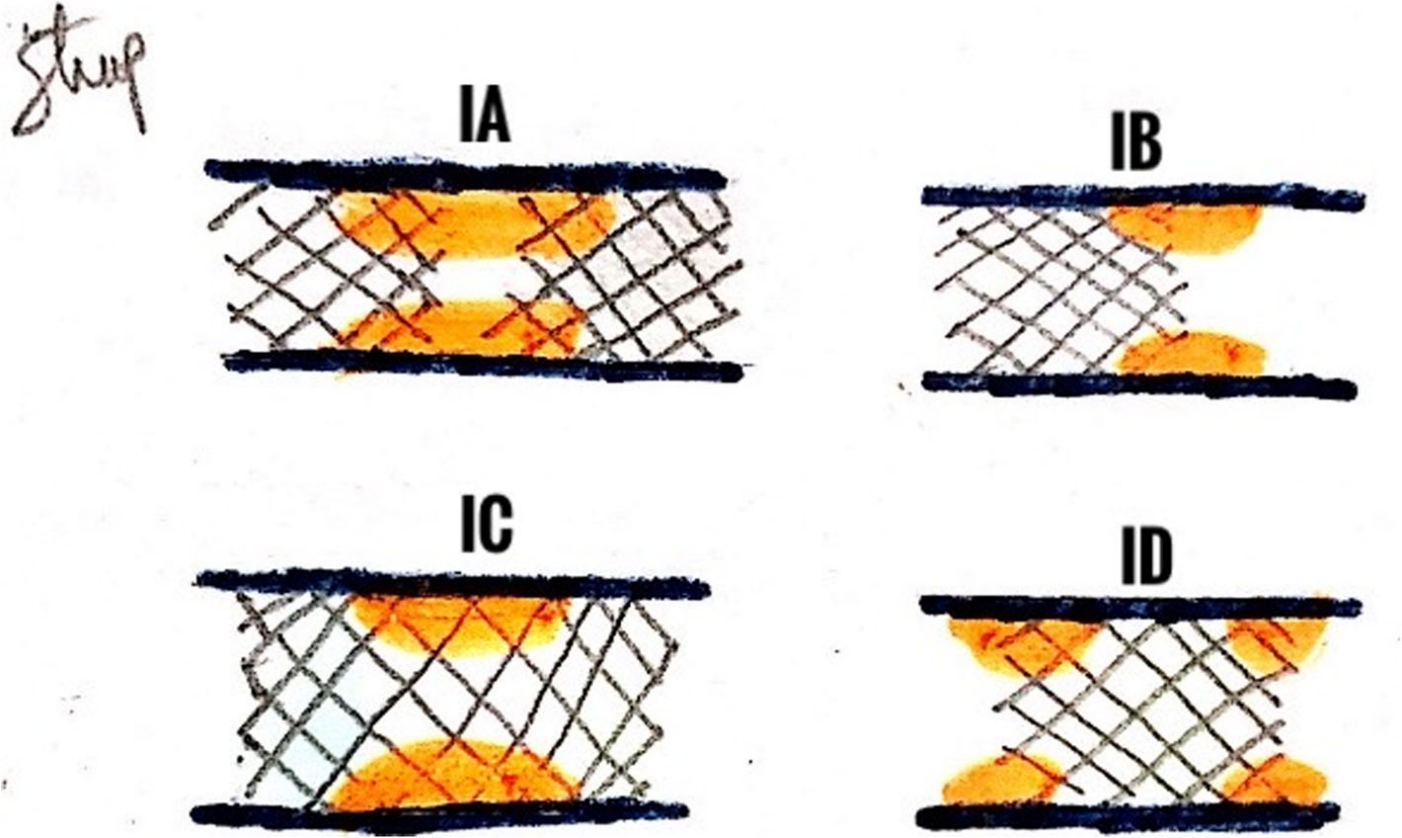
Focal ISR classification according to angiographic classification. IA Articulation or gap. IB Margin. IC Unifocal. ID Multifocal

Angiographically, restenosis defines an obstruction of > 50% of the luminal diameter at the site of intervention segment (or within the stent) or within the adjacent 5 mm from the intervention segment after PCI procedure (Fig. 1) [13–15]. It is caused by an endothelial pathologic response to the injury. The harmful vascular remodeling and neointimal proliferation of vascular smooth muscle cells and/or new occurring atherosclerotic process is called “neoatherosclerosis.” Restenosis usually is managed with immediate reintervention either by coronary artery bypass graft (CABG) or another PCI. Some cases found restenosis accidently while performing coronary angiography in asymptomatic patients [15]. Many studies report that restenosis (Fig. 1) incidences occur in more than 30–40% population after PCI procedure [14–16].

There are numerous ways to diagnose luminal obstruction after PCI, such as comparing minimal luminal diameter (MLD) post-intervention with follow-up diameter, intravascular ultrasound (IVUS), and noninvasive technique like optical coherence tomography (OCT), multidetector computer tomography (MDCT), and fractional flow reserve (FFR) [14]. Noninvasive imaging modality was more preferred by the patient, but its role in detecting restenosis is still limited [17].

A previous retrospective study with 3448 patients found that independent risk variables for restenosis are history of smoking, diabetes, ostial lesion, bifurcation lesion, post-procedural MLD, reference diameter (less than 3.25 mm), and lesion length (> 30 mm) [18]. Other studies concluded that restenosis depends on several predictors such as clinical, procedural, and angiographic [16, 19].

Right after inflating the balloon, the entire thickness of the artery will stretch. Tunica media and adventitia layer might get injured by the balloon’s expansion, and the healing process occurs following the vascular injury. The healing process consists of several processes such as platelet aggregation, inflammatory cell infiltration, release of growth factors, medial smooth muscle cell (SMC) modulation and proliferation, proteoglycan deposition, and extracellular matrix (ECM) remodeling [14]. The satisfying end point of the healing process is reendothelialization without significant diameter narrowing.

In pathogenic conditions, neointimal hyperplasia as a negative vascular remodeling process occurs and it causes vascular narrowing. Nitric oxide releases right after stress injury to the endothelial wall. In this situation, cells also release some cytokines like platelet-derived growth factor (PDGF) and transforming growth factor-β (TGF-β). Those cytokines promote migration and proliferation of vascular smooth muscle cells (VSMC) [14]. Besides cellular inflammatory response (such as infiltration of macrophage, T cell, B cell, and eosinophil), patient’s specific risk factor (clinical and genetic) and procedure-related factor also contribute to restenosis event [20]. There are some clinical conditions related to restenosis event such as diabetes mellitus, smoking status, hypertension, myocardial infarction, previous PCI, and female gender [16, 20].

### Drug-eluting balloon (DEB) VS stent-based PCI

Historically, the use of stent implantation aims to deal with vascular dissection [21]. Nonetheless, long-term benefits of stent implantation for coronary artery disease are still debatable [22]. On the other hand, DEB has been proven to elude the late complication of the coronary restenosis as well as simplifying PCI procedure. Two studies from the Belgian Netherland Stent Study (BENESTENT) and Stent Restenosis Study (STRESS) conclude only 5.1% and 6.9%, respectively, balloon-based angioplasty patients need to undergo crossing over to stent technique due to acute vessel complications [21]. In the stent-based PCI era, there are several indications for using balloon-based angioplasty such as stent delivery failure/expected to be difficult due to calcification and intervention for in-stent restenosis/stent thrombosis. Instead, head-to-head comparison between balloon-based angioplasty and stent-based angioplasty in small vessel displays DEB is not inferior compared to the stent-based PCI in terms of re-stenosis’ rate [22]. Furthermore, intracoronary stent restenosis (ISR) and stent thrombosis remain the major problems even with drug-eluting stent (DES) techniques [21, 23, 24]. The ISR incidence is still as high as 12% after 6 months follow-up even with the new generation of the eluting stent [25].

The DEB is an angioplasty procedure using semi-compliant balloons layered with a cytotoxic chemotherapeutic drug. It can spread the drug more equivalently to the endothelial lining compared to the DES technique [26]. One study using a sirolimus-coated balloon indicates low major adverse cardiac event (MACE) rate (5.38%) within 12 months follow-up [21]. A single-center registry study (DEB = 601 patients and DES = 1594) reveals that balloon angioplasty is more beneficial to use in patients with more than three vascular diseases and smaller vessels and finds that the stent-based group favors stent/lesion thrombosis and targets vessel re-infraction after > 1 year follow-up [27]. In other advantages regarding medication therapy, many cardiologists are only using a single antiplatelet for 1 month after DEB procedure compared to stent technique that requires dual antiplatelet therapy for around 1 year [21, 25, 27].

Other studies (25 patients) also discovered potential benefits of DEB that could let the blood vessel to reclaim its initial vasomotion ability compared to the stent-based PCI [28]. During blood vessel remodeling after angioplasty, the metal stent could be in malposition [29]. A long-term study comparing the MACE rate from DEB only with paclitaxel-coated balloon and DEB + bare metal stent (BMS) in a small coronary artery concludes low MACE rate in DEB-only angioplasty (6.1% vs 37.5%) after a 3-year follow-up study [30]. A multi-center international registry also favors paclitaxel-coated balloon in small vessels with only a small number of undesirable events (3.6% target lesion revascularization/TLR, 0.6% had vessel thrombosis in non-target lesions, no cardiac death report or coronary artery bypass graft surgery) [31]. A long-term longitudinal study up to 9 years follow-up shows that balloon angioplasty is more superior compared to BMS and DES in terms of target vessel re-infraction caused by restenosis and also lesion thrombosis [21].

Many randomized controlled trials support evidence that DEB is a better option for lesions in small coronary vessel bifurcation lesions, long lesions, and pediatric interventions [25]. The BMS and DES group are dealing with some problems such as metallic cage which could delay reendothelialization, malposition, under-expansion, and chronic inflammation that may contribute to restenosis [21]. Many of the preceding studies display promising results of the DEB exclusively in small coronary arteries, but recent international consensus emphasize the DEB even in large vessel disease and high bleeding risk patient [31]. The DEBUT trial (de-novo coronary artery lesions in patients with high bleeding risk) includes a larger diameter of coronary arteries lesions (2.5–4 mm) patient in the trial and only 1% MACE incidence at 9 months follow-up compared with 14% in the bare metal stent group (risk ratio 0.07 [95% CI 0.01 to 0.52]) [32]. In another single-center study in which 60% of the patients with lesions in more than 3 mm coronary vessels and de novo lesions including large proximal coronary arteries are managed by DEB, it shows a 7.1% MACE rate in stable coronary artery diseases and 12% in acute coronary syndrome [33].

### Limitation of DEB

Despite all the benefits of DEB, still, it has some limitations in specific scenarios. A recent study comparing DEB and DES for management of ISR in DES (1377 lesions) and BMS (722 lesions) concludes that DEB for DES-IR treatment have a tendency of higher risk of ischemia-driven TLR (HR 1.67; 95% CI 1.21 to 2.31) and target vessel revascularization (HR 1.39; 95% CI 1.05 to 1.84) compared to DES in 3 years follow-up [34]. A randomized clinical trials with 1177 patients and shorter end point (1 year follow-up) shows DEB associated with higher risk of MACE, target vessel revascularization, TLR, binary stenosis, and more diameter stenosis % in the DES-ISR group compared to the new generation DES [35]. The New Tokyo Registry consists of as much as 304 consecutive patients (333 lesions) comparing DEB in the management ISR based on the number of metallic layers [36]. The study consists of three groups: 1 stent layer (166 patient), 2 stent layers (87 patient), and 3 and more stent layers (51 patients). It suggests that 3 and more stent layers is an independent predictor for MACE compared to 1 stent layer (HR 2.21; 95% CI 1.12–4.36). Unfortunately, the study about DEB and DES-ISR is limited. A multi-center study from Korea proves that optimizing procedure-related factors during DEB for management of DES-ISR could be the answer [37]. Optimize preparation includes 1:1 balloon and vascular diameter, and longer DEB inflation time more than 60 s could bring a magnificent result in a 1-year follow-up.

Regarding dual antiplatelet therapy (DAPT) in DEB patients, the European Society of Cardiology (ESC) recommends 3 months in using DAPT [38]. However, a study which compares about superiority combination DEB and DAPT and other modalities is not clearly been done before. The ABSORB III study compares 1322 patients with bioresorbable vascular scaffold (BVS) and DES, from a 1-year study BVS non-inferior than DES. However, a 3 years follow-up shows BVS turn in higher TLF incidence than DES [39]. A study comparing head-to-head between BVS and DEB is limited.

Transplant coronary artery disease (TCAD) is also one of the major issues faced by both the cardiac interventionist and cardiac surgeon [40]. The TCAD incidence is quite high, as much as 30–50% within 5 years [41]. However, there is no study about TCAD management with DEB technique. Everolimus as immunosuppression therapy in heart transplant could give benefits in long-term TCAD incidence, but cannot eliminate it [42]. The current understanding about TCAD is not only by immunologic respond but also non-immunologic respond [43]. Still, recurrent TCAD becomes frustrating event in heart transplant, and palliative PCI might be the best management which could be done right now.

There are three major parts of stent: a metal scaffold, active pharmacological agent, and drug’s carrier. Latest generation of stents has thinner struts, and those models correlate with less restenosis incident [44]. The Triple Assessment of Neointima Stent FOrmantion to Reabsorbable polyMer With Optical Coherence Tomography (TRANSFORM-OCT) analyzed a multivessel disease which compares new generation (thinner struts) BMS and DES [45]. Both newer generation of DES and BMS shows magnificent results in terms of neoatherosclerosis at 18 months end point. Besides, in terms of thick-thin struts, polymer coating technology surface (smooth surface, microporous, microporous, nanoporous, and drug-filled stents) also helps to increase drug delivery and decrease ISR problem [46]. A recent update about biotechnology of stent’s scaffold might diminish DEB function in the future; even third generation of DES could overcome ISR in diabetic patients which has high rate of ISR [47].

## Conclusion

PCI was one of the revolutionary technologies in coronary artery diseases. The indications for PCI technologies are evolving parallel with new studies which show the beneficial use of PCI. Nowadays, stent-based techniques are the most common procedures for patients with de novo artery coronary disease. However, not all cases could be done by stent-based PCI. The DEB techniques have a considerable potential on that case. Currently, a growing clinical indication for de novo coronary artery lesion is that it can be managed by DEB-only PCI since it is superior in long-term effect compared to either BMS or DES. The restenosis rate of the DEB is lower compared to stent-based PCI. Additionally, DEB-only PCI could offer other benefits in terms of cost, implantation technique, and antiplatelet regiment. Nowadays, many studies start to use DEB-only PCI in a larger blood vessel diameter and it shows promising result. On the other hand, DEB also has some limitations especially for the management of DES-ISR. Unfortunately, a head-to-head study of DEB and other methods is limited and needs further investigation.

## Data Availability

Not applicable

## Abbreviations

CAD: Coronary artery disease
PCI: Percutaneous coronary intervention
NHLBI: National Heart, Lung, and Blood Institute
PTCA: Percutaneous transluminal coronary angioplasty
OMT: Optimal medical therapy
NSTEMI: Non-ST elevation myocardial infarct
STEMI: ST elevation myocardial infarct
CK-MB: Creatinine kinase myocardial band
GRACE Score: The Global Registry of Acute Coronary Events Score
ACS: Acute coronary syndrome
CABG: Coronary artery bypass graft
MLD: Minimal luminal diameter
IVUS: Intravascular ultrasound
OCT: Optical coherence tomography
MDCT: Multidetector computer tomography
SMC: Smooth muscle cell
ECM: Extracellular matrix
PDGF: Platelet-derived growth factor
TGF-β: Transforming growth factor-β
VSMC: Vascular smooth muscle cells
BENESTENT: Belgian Netherland Stent Study
STRESS: Stent Restenosis Study
ISR: Intracoronary stent restenosis
DES: Drug-eluting stent
MACE: Major adverse cardiac event
BMS: Bare metal stent
DEBUT trial: De-novo coronary artery lesions in patients with high bleeding risk
